# Digital Holographic Microscopy: A Quantitative Label-Free Microscopy Technique for Phenotypic Screening

**DOI:** 10.2174/13862073113166660062

**Published:** 2014-01

**Authors:** Benjamin Rappaz, Billy Breton, Etienne Shaffer, Gerardo Turcatti

**Affiliations:** Biomolecular Screening Facility (BSF), Ecole Polytechnique Fédérale de Lausanne (EPFL), Lausanne, Switzerland

**Keywords:** Cell-based assay, differential interference contrast, digital holography, ion channel, high-content screening, image-based screen, phase contrast, phenotypic drug discovery.

## Abstract

Digital Holographic Microscopy (DHM) is a label-free imaging technique allowing visualization of transparent cells with classical imaging cell culture plates. The quantitative DHM phase contrast image provided is related both to the intracellular refractive index and to cell thickness.

DHM is able to distinguish cellular morphological changes on two representative cell lines (HeLa and H9c2) when treated with doxorubicin and chloroquine, two cytotoxic compounds yielding distinct phenotypes. We analyzed parameters linked to cell morphology and to the intracellular content in endpoint measurements and further investigated them with timelapse recording. The results obtained by DHM were compared with other optical label-free microscopy techniques, namely Phase Contrast, Differential Interference Contrast and Transport of Intensity Equation (reconstructed from three bright-field images). For comparative purposes, images were acquired in a common 96-well plate format on the different motorized microscopes.

In contrast to the other microscopies assayed, images generated with DHM can be easily quantified using a simple automatized on-the-fly analysis method for discriminating the different phenotypes generated in each cell line. The DHM technology is suitable for the development of robust and unbiased image-based assays.

## INTRODUCTION

Phenotypic screens [1-3] rely on image-based cellular assays for analyzing cell morphological variations or alterations. For these applications, it is of paramount importance to use the least invasive imaging techniques possible to avoid phenotypic changes induced by the imaging process itself. Together with the costs of labeling agents, this is a fundamental motivation for favoring non-phototoxic label-free imaging techniques [4-6].

Unfortunately most cells are transparent or generate only modest changes in the amplitude of light, which makes them hard to image with the simplest label-free optical imaging techniques such as bright field microscopy. Those transparent specimens, on the other hand, generally alter—or shift—the phase of light more significantly. Examination of such phase objects has led to the development of optical contrast-enhancing imaging techniques like phase contrast (PC), initially proposed by Zernike [7], as well as Nomarski Differential Interference Contrast (DIC) [8]. These methods do not readily provide quantitative information on the specimen-induced phase shifts. Furthermore, their inherent contrast mechanism and related artifacts [9] make automated cell segmentation very difficult and hardly robust.

Recently many companies have been integrating in their automatized microscopes an imaging mode based on Transport of Intensity Equation (TIE) [10, 11] generating pseudo-quantitative phase contrast images from a z-stack of at least three bright-field images. TIE imaging—or digital phase contrast—can be implemented on any bright field imaging system equipped with a motorized z-stage. In practice, these images are mostly used to segment cells but they can also serve as data source for direct analysis.

In addition to the above-mentioned microscopy techniques, the availability of lasers, modulators and sophisticated detectors (in many cases CCD cameras) has promoted the development of various interferometric techniques. Unlike PC and DIC, interferometric techniques present the great advantage of quantitatively measuring optical parameters like the phase shifts introduced by transparent specimens, a parameter that is related to the morphology and intracellular content of the cell [9, 12]. One such noninvasive and label-free interferometric technique is digital holographic microscopy (DHM). Since the DHM signal is related to biophysical parameters, it can reveal absolute cell volume [12], dry mass [13], protein concentration [14], transmembrane water influx/outflow and permeability [15] and cell death [16, 17], therefore making this noninvasive imaging technology suitable for sensitive measurements of various cellular events, such as cell migration, proliferation, death and differentiation.

Suitability of DHM for image-based screening in multiwell cell culture plates has recently been confirmed [16]. In order to assess the ability of DHM to distinguish distinct phenotypes we measured the Z’-factor [18] obtained on two representative cell lines, HeLa (round shape and high contrast) or H9c2 (elongated and flatter shape, low contrast) treated with two cytotoxic compounds: doxorubicin triggering cell death assessed by a rounded phenotype; or chloroquine producing autophagy, a phenotype showing small vesicles. Endpoint measurements at 24 h and time-lapse recording were used to investigate morphological changes and quantify the effect of the compounds on the assayed cells (toxicity, EC_50_, onset of the response, etc.). Finally, we compared DHM endpoint results with those obtained using other label-free microscopy techniques (DIC, PC and TIE).

## MATERIAL AND METHODS

### Cell Culture

HeLa (ATCC^®^, CCL-2™) and H9c2 (ATCC^®^, CRL-1446™) cells were maintained in Dulbecco's modified Eagle's GlutaMAX medium (Life Technologies Ltd., ref. 32430) supplemented with 10% irradiated and heat inactivated fetal bovine serum gamma (Life Technologies Ltd., ref. 10101-145), and were grown at 37°C in 5% CO_2_ with ~95% relative humidity. Before drug treatment, cells were trypsinized, seeded in 96-well BD-falcon imaging plate (ref. 353219) at a density of 3000 (H9c2) or 6000 (HeLa) cells per well and grown for 24 hours. Cells were at 25-60 % confluency at the time of measurement.

### Drug Treatment

After the 24 h growth period in the incubator, cells were treated with doxorubicin hydrochloride (Sigma-Aldrich, ref. D1515) or chloroquine diphosphate salt (Sigma-Aldrich, ref. C6628) at 30 µM. The control was the drug vehicle (0.3% DMSO in PBS for doxorubicin and PBS for chloroquine). After overnight incubation in culture medium at 37°C/5% CO_2_, cells were fixed with paraformaldehyde 4% (PFA) and rinsed 3× with PBS before image acquisition. Control DHM images acquired before and after fixation showed no PFA effects on the parameters measured (data not shown). For time-lapse measurement, serial dilutions of the compounds were added to the cells 24 h after plating.

### Image Acquisition

PFA-fixed cells were imaged at room temperature (~22°C). DHM time-lapse measurements on live cells were achieved in a Chamlide WP incubator system for 96-well plate (LCI, South Korea) set at 37°/5% C02 with high humidity. Time-lapse images were acquired each 10 minutes for 24 h.

For each experiment, four images per well were acquired and the corresponding measurements were averaged to yield a mean value per well.

DHM images were acquired in an off-axis configuration on a commercially available DHM T-1001 from LynceeTec SA (Lausanne, Switzerland) equipped with a motorized xy stage (Märzhäuser Wetzlar GmbH & Co. KG, Wetzlar, Germany, ref. S429). Images were recorded using a Leica 10×/0.22 NA objective (Leica Microsystems GmbH, Wetzlar, Germany, ref. 11506263).

Differential Interference Contrast (DIC) and Phase Contrast (PC) images were acquired on a Zeiss Axio Observer Z1 (Carl Zeiss AG, Jena, Germany) equipped with an APlan 10×/0.25NA Ph1 objective with a Ph1/0.55NA condenser for PC and an APlan 10×/0.25NA with a DIC analyzer II for DIC.

Bright-field images were acquired on an Operetta system (Perkin Elmer, Waltham MA, USA) equipped with a 10×/0.25NA objective and a 740 nm LED light source. The three Z-positions were -5; 0 (focused) and +5 µm. TIE images were reconstructed from these BF images using equations published in ref. [11].

Finally, DHM, PC, DIC and BF images were all acquired from the same field.

### Label-Free DHM Technology

Briefly, DHM [9, 16, 19] is a label-free interferometric microscopy technique which provides a quantitative measurement of the optical path length (OPL, related to the optical density of the cell). It is a two-step process where a hologram consisting of a 2D interference pattern is first recorded on a digital camera and the contrast (phase) images are reconstructed numerically using a specific algorithm [9]. The (phase) contrast in DHM images is quantitatively related to the optical path difference (OPD), expressed in terms of physical properties as:

(1) $$\mathrm{OPD}\left(x,y\right)=d\left(x,y\right).\left[\bar{n_{c}}\left(x,y\right)-n_{m}\right],$$

where *d(x,y)* is the cell thickness, *n_c_(x,y)* is the mean *z*-integrated intracellular refractive index at the *(x,y)* position and *n_m_* is the refractive index of the surrounding culture medium. Simply put, Eq. (1) means that the OPD signal is proportional to both the cell thickness and the intracellular refractive index, a property linked to the protein and water concentration of the cells [12, 13].

DHM systems generally use a low intensity laser as light source for specimen illumination and a digital camera to record the hologram. Here, the 684 nm laser source delivers roughly 200 µW/cm^2^ at the specimen plane— that is some six orders of magnitude less than intensities typically associated with confocal fluorescence microscopy. With that amount of light, the exposure time is only 400 µs. An extensive quality control of DHM can be found in [14].

### Cell Count and Confluency

For each microscopy technique the number of cells was similarly measured in ImageJ (Wayne Rasband, NIH). Images were first blurred with a Gaussian filter of 3 pixels (1.86 µm) and cells were then counted using the “Find Maxima” function.

Confluency was measured by first thresholding the images with a pre-determined value to obtain a mask and then by measuring the surface ratio of the mask to the total area of the field of view.

Analysis is independent of cell seeding density as DHM is capable of segmenting cells at different degrees of confluency [16, 20]. OPD is stable for a wide range of cell confluencies (see supplementary Fig. **3**).

### Image Segmentation and Data Analysis

With DHM images, phenotypic changes were quantified by using two distinct analysis workflows: direct raw OPD measurement and image analysis performed with CellProfiler analysis (CPA).

### DIC and PC Image Restoration

DIC and PC contrasts are generated through a well-known pattern of interference in the microscope optical path. Therefore, by knowing the characteristics of the objective and microscope optical path, it is possible to deduce (or restore) the optical path length difference (OPD) of the recorded specimen [21-25]. We used the algorithm published in ref. [21] to reconstruct DIC images and the algorithm published in ref. [22] to reconstruct PC images. As some of the parameters required by the PC algorithm are proprietary to the MO manufacturer (for instance the width and distance of the phase ring inside the MO) we used trial and error to estimate the best values. Finally, we compared the quantitative capability of PC- and DIC-restored OPD images using the same workflow used to analyze DHM images (described in the following sections).

### Average OPD Measurement

The total OPD value is obtained by adding the OPD value recorded in each of the (*x,y*) masked pixel of the image (obtained to measure the confluency, see above). The average OPD is obtained by dividing the total OPD by the surface of the mask and is a measure of the optical density of the cells normalized by the confluency. This value is dependent on the cell shape (it increases with rounded cells) and is independent of cell confluency. Average OPD is an unbiased parameter that can be used to categorize phenotypes [16].

### CellProfiler Analysis

**Fig. (2):**
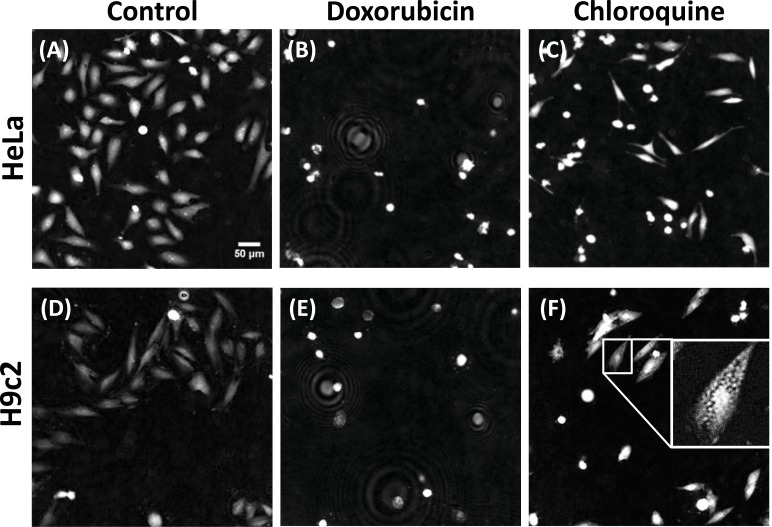
Compound induced phenotypes. Representative endpoint (24 h) images of HeLa (A, B, C) and H9c2 (D, E, F) cells in control condition (A, D) treated with 30 µM doxorubicin (B, E) or 30 µM chloroquine (C, F). Scale bar: 50 µm. All images are drawn with the same intensity levels (except inset image). Ring pattern in the doxorubicin images are dead cells detached from the well-plate.

For each microscopy technique, image segmentation quantification and analysis was performed using the open-source software CellProfiler (Broad Institute, MA, http://www.cellprofiler.org/, r11710) [26]. CellProfiler is able to successfully detect, segment and analyze individual cells in DHM images [16]. DHM, TIE, PC and DIC restored images were processed using the same CellProfiler pipeline (with only slight modifications to account for signal intensity differences). Training (with CellProfiler Analyst and machine-guided learning) was performed separately for each imaging technique. Three object classes and the related main criteria to populate the training sets were defined as follows: control (untreated cells, elongated and well attached cells, see Fig. **2**), “round” (round and intense cells – used for cells treated with doxorubicin, see Fig. **2**) or “vesicles” (less attached cells, small and round vesicles present – used for cells treated with chloroquine, see Fig. **2**) and segmentation 

error objects. Classification was based on a selection of parameters (including intensity, texture, granularity, area and shape) measured by CellProfiler. Results are presented as number of cells expressing the round or vesicle phenotypes divided by the total number of cells minus the number of segmentation errors objects.

### Statistical Comparison

For statistical analyses, the mean value and the standard deviation for each parameter (avg. OPD and “round”/ “vesicle” phenotypes) were measured from 12-16 different wells (mean of 4 fields of view) for each condition. These values were then used to calculate the Z’-factor [18] for each condition (cell type and phenotype). A value close to 1 indicates an excellent screening window whereas a value below 0.5 reflects a marginal essay. It can be argued that this statistical parameter is not the best criteria for the assessment of the quality of a screen concerning image-based assays [27]. However, the Z’-factor is appropriate for relative comparisons of different read-outs technologies like in the present study where the different microscopic techniques were tested under the same experimental conditions and evaluated using similar or identical analysis methods.

### Time-Lapse Measurement

For time-lapse measurement, the area under the curve of the direct measurement of average OPD or the percentage of the “round” or “vesicle” phenotype calculated with CellProfiler Analyst were measured with Prism6 (GraphPad) and used to determine the dose response parameters for each compound and cell type using a least squares minimization of variable slope dose-response curves (with four parameters). Control data points defined as the vehicle without drug were arbitrary plotted at 2 logs under the lowest concentration.

## RESULTS AND DISCUSSION

Digital holographic microscopy is a label-free microscopy technique in which contrast is related to biophysical parameters (notably cell thickness and refractive index distribution) [9]. It has been shown to be capable of revealing absolute cell volume [12], dry mass [14], protein concentration [13], transmembrane water influx/outflow, and permeability [15, 17], thus making this noninvasive imaging technology suitable for sensitive measurements of various cellular events, such as cell migration, proliferation, death, and differentiation. Applications of DHM for monitoring cellular responses have already been extensively reported [9, 15, 17, 20, 28, 29] and its potential for cytotoxicity assessment has recently been validated through comparisons with fluorescence methods [16, 17] or electron microscopy [30]. Here, we rely on DHM contrast measurements to highlight the morphological and local biomolecule concentration changes (proteins, nucleic acids, etc.) induced during drug treatment.

To test the ability to distinguish various phenotypes for applications in phenotypic drug discovery, we first imaged HeLa (rhomboid shape and high contrast) and H9c2 (elongated and flatter shape, low contrast) cells treated for 24 h with two drugs chosen for the phenotypes they produce: doxorubicin, a drug used in cancer chemotherapy, which produces round cells of higher contrast, representative of a dying cell phenotype; and chloroquine, a drug used for the treatment or prevention of malaria which produces small vesicles, representative of an autophagic machinery defect phenotype. Representative images (Fig. **2**) illustrate the different phenotypes produced by the interfering compounds selected for this study.

### Approaches to Extract Information from DHM Images

The different conditions yielded distinct qualitative differences which were quantified using the three following approaches (described in the methods section and summarized in Table **1**): cell count and confluency, average OPD and CellProfiler Analyst.

### Cell Count and Confluency

**Fig. (3):**
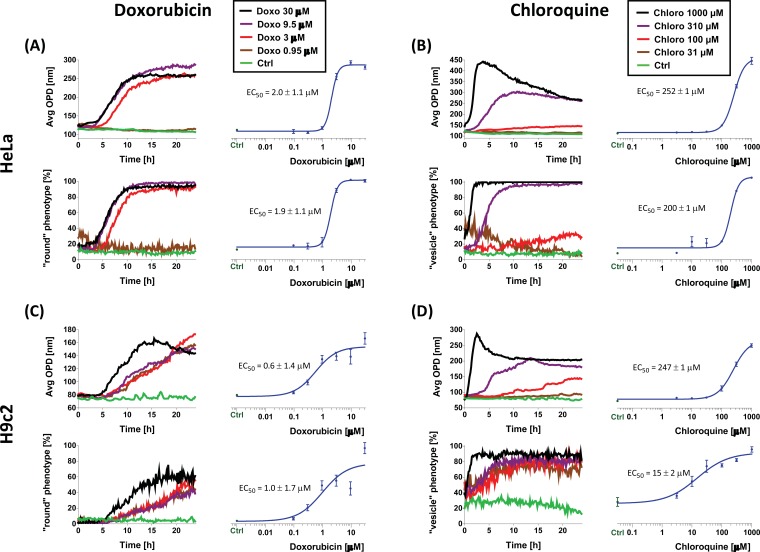
Time-lapse measurements. HeLa (A, B) and H9c2 (C, D) treated with serial dilution of doxorubicin (A-C) or chloroquine (B-D) were imaged each 10 min for 24 h. For each condition, the average OPD and the percentage of round phenotype was measured. Doseresponse graphs (in blue) are area under the curve calculated from the graph on their left.

The first parameters, cell count and confluency, showed a marked decrease due to cell death and proliferation inhibition (supplementary Table **1**). However, the variability on cell count and confluency measured by the standard deviation between wells was too high to yield a Z’-factor compatible with high-content screening (HCS) applications. It should be noted that the values obtained with HeLa were generally better (*i.e.* higher Z’ values) compared to those obtained on H9c2 principally due the higher contrast of HeLa cells. H9c2 cells have a flatter shape and thus a lower signal (about half the OPD signal, Fig. **3** “ctrl”) which resulted in a higher noise level and less precise results.

Anyways, cell count and confluency are not the best suited parameters to distinguish subtle phenotypes or conditions affecting only the morphology of the cells and not their number, therefore the need of more advanced metrics described below.

### Average OPD

One way to make cell confluency a more powerful parameter for phenotypes distinction is to combine it with a measure of the changes in the DHM signal. This parameter measures the “Average OPD”: the mean OPD value over the total image surface occupied by cells. This represents an equivalent of the total dry mass present in the field of view [13]. This value increases with cell growth and is independent of cell shape.

We have used this new parameter to measure the changes in the surface normalized average optical path length difference upon drug application. This parameter measured on the whole population of cells was found to efficiently discriminate all the conditions tested (see Table **1**).

Average OPD recording is obtained in an unbiased manner without human intervention and can be fully automatized. This measurement is obtained as the images are reconstructed, which can be in real-time, and are readily available for fast phenotypic classification with no further calculation steps required. Average OPD is the analysis of choice when tests assessing general morphological changes need to be performed rapidly on a large number of samples (*e.g. *in primary screen). While average OPD represents a very powerful factor, it remains a unique, global parameter based on broad images and not individual-cell. When subtle phenotypes need to be distinguished, especially those only mildly affecting the average phase signal or when sub-populations need to be categorized, a multiparametric individual-cell approach may be more suitable.

### CellProfiler Analyst (CPA)

In order to distinguish subtle phenotypes, an individual-cell multiparametric analysis, like CellProfiler Analyst (CPA), will be the method of choice. Z’ values for each of the analysis approaches are given in Table **1**.

### Time-Lapse Measurements

Phenotypes evolve with time, therefore measuring the onset and following their evolution with time-lapse measurements could provide much more information than simple endpoint measurements, in particular for hit validation and characterization. Furthermore, time-lapse measurements offer the possibility of using each and every well as its own negative control, provided that the imaging process is initiated before addition of compounds, further reducing inter-well variability and increasing measurement precision. As DHM is label-free and noninvasive, it is particularly well suited for these kinds of experiments.

We have recorded HeLa and H9c2 cells treated with serial dilutions of doxorubicin or chloroquine for 24 h (1 image every 10 mins). Average OPD and CPA analysis were performed on each data set and plotted versus time. EC_50_ values were obtained by measuring the area under the curve of the DHM and CPA data and fitting them with a sigmoid curve. The area under the curve is a better measurement than simple endpoint measurement because it integrates all the changes that have occurred during the recording period and also averages the contribution of potential outliers, thus providing a more accurate readout. Measured EC_50_ values are in good agreement with those found in the literature [31, 32].

As shown in Fig. (**3**), EC_50_ values obtained from raw average OPD measurement and CPA can present some variations, which can be explained by the difference in the analysis approaches. For instance with H9c2 a medium concentration of chloroquine induces the formation of autophagic vesicles whereas a higher concentration will eventually trigger apoptosis or necrosis. Average OPD will be more sensitive to the morphological changes due to necrosis/apoptosis yielding a higher EC_50_ value (247 versus 15 µM) compared with the same cells monitored with the CPA analysis for the formation of vesicles (which occurred with lower concentration of chloroquine). Indeed OPD only reacts to average changes of OPD (and responds to cellular rounding) whereas CPA (trained to recognize both round cells and vesicles) will provide an earlier signal as soon as vesicle formation occurs (earlier than the rounding of the cells). These values closely match those found in the literature [32]. On the contrary, HeLa cells have much smaller vesicles which are less well detected with CPA analysis, thus explaining the closer EC_50_ values measured. Therefore, although similar, average OPD and CPA analyses provide slightly different and complementary results.

In addition, time-lapse allowed measuring the early onset of the phenotype by looking at the first time-point which deviates significantly from the baseline. For instance, we observed that doxorubicin induces a rounded phenotype after 4 hours with HeLa cells and 5 hours with H9c2 cells (see Fig. **3**) whereas the vesicle phenotype, in chloroquine-treated cells, occurs much faster and can already be observed at the first time-point (about 5 minutes after drug addition). Finally, recording of the average OPD over time provides additional information on the future viability of the cell [17]. This is illustrated for example in the case of the application of a high dose of chloroquine (Fig. **3** and supplementary Fig. **4**). In this case, the average OPD signal decreases after reaching a peak, whereas in Lactate Dehydrogenase Activity Assay (a cellular membrane integrity indicator) the maximum signal is obtained after this average OPD peak, confirming that decrease of the OPD signal is an indication of cell lysis as a consequence of compromised membrane permeability. In conclusion, both raw average OPD and CPA data can be used to measure EC_50_ values in a noninvasive way either at endpoint or during time-lapse experiments.

### Imaging Mode Comparison

DHM is often compared to Phase Contrast (PC) and Differential Interference Contract (DIC) microscopy as these optical techniques are also label-free and require only a low light dose to generate contrast images from transparent cells. In practice, however, PC and DIC are mostly used for visual inspection to assess cell confluency or qualitative morphological aspects.

We have compared DHM with DIC, PC and also TIE (which is obtained from a z-stack of 3 bright-field images which can easily be recorded on any microscope). We specifically examined the quantitative information that can be extracted in each of these microscopy techniques and how it can be relevantly used to distinguish phenotypes in a HCS context.

Fig. (**4**) shows representative images of the same field obtained in DIC, PC, DHM, bright-field (BF) and TIE. DIC and PC images highlight fine details (like vesicles and cell extensions), but their respective mechanisms for contrast generation are not compatible with most common automated cell segmentation algorithms, which very often rely on a thresholding of some sort. Indeed, the contrast fluctuations over the cell, illustrated by the profiles drawn in the lower panels of Fig. (**4**), prevent cell segmentation. DIC, PC and BF produce strong signals at cell edges but cell height cannot be measured. On the contrary, DHM and TIE signal is proportional to cell height and can therefore directly provide morphological data and easily-segmentable images.

Although some information is permanently lost with DIC and PC (like the information parallel to the shear axis in DIC [9]), many attempts have been conducted to take advantage of the details provided by these techniques to restore morphological data from their raw signal [21-24]. Using published algorithms we extracted the OPD data from DIC and PC images (see supplementary Fig. **1**) and then used the same DHM workflow (direct raw OPD analysis and CPA analysis) to measure Z’-factors.

The PC restoration algorithm used provided good results for certain shapes of cells but not for others. For instance optimization of the parameters yielded good results for flat cells but not for rounded cells (see supplementary Fig. **1**) and thus could not be automatized for analysis and was not further investigated. On the other hand the algorithm used to restore DIC images worked for all cell shapes and phenotypes with only mild artifacts (like the shadow-tail seen in supplementary Fig. **1**) and could thus be automatized for analysis.

TIE images were similarly compared using the same analysis workflow.

**Fig. (4):**
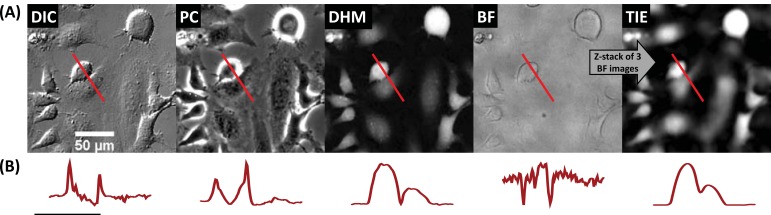
Imaging mode comparison. (A) Differential Interference Contrast (DIC), Phase Contrast (PC), Digital Holographic Microscope (DHM), Bright-field (BF) and Transport of Intensity Equation (TIE, generated from a z-stack of 3 BF images) from the same field of view. (B) normalized profile along the red path in DHM, DIC, PC, BF and TIE images illustrating the quantitative aspect of DHM and TIE and qualitative aspect of the other techniques. Horizontal scale bar = 50 µm, vertical scale bar = 200 nm OPD (DHM); arbitrary units (DIC, PC, BF and TIE).

DIC restored images can only provide usable data (Z’-factor well above 0) when measuring the most obvious phenotype (“round”, doxorubicin-treated) with HeLa cells having the highest contrast (highest signal-to-noise ratio). In contrast, TIE images yielded satisfactory Z’-factors for most of the phenotypes, but required a motorized z-stage and the combination of 3 images, limiting high-speed applications and measurement of phenotypes producing rapid morphological changes. Additionally, the lower level of details present in TIE images (Fig. **4**) could impair some phenotypes identification. These two points constitutes a considerable disadvantage of TIE, when compared with DHM.

In addition, the water meniscus that forms in the well also degrades the quality of PC and DIC images (see supplementary Fig. **2**) hence preventing a direct interpretation of these images for HCS applications and seriously impairing time-lapse measurement due to meniscus formation caused by evaporation over time. The need to acquire three BF images for TIE also prevents the imaging of fast changing phenotypes.

### Advantages and Limitations of DHM for Phenotypic Drug Screening

Images obtained with DHM are quantitative in an absolute manner, meaning that they are totally independent of microscope hardware (considering an identical light source wavelength) and acquisition settings and do not require any calibration. In addition, the cellular OPD contrast is directly related to the cell thickness, which facilitates the isolation of individual cells by simply using a threshold-based algorithm, which is not possible with raw BF, PC or DIC images.

We recently showed that DHM could be efficiently used for screening applications in multi-well culture plates [16]. The digital re-focusing feature of digital holography allows performing measurements on a setup not equipped with a motorized z-stage and accelerates the recording process, as no mechanical focusing step is required.

DHM imaging and screens are routinely performed in 384-well plates but for comparative purposes with other techniques during this work, images were acquired in a common 96-well plate format. We typically image a 96-well plate recording four images per well in less than 4 minutes. The speed of image acquisition is only currently limited by the speed of the xy stage-motors.

Measuring simple parameters such as culture confluency and cell count can easily be achieved by DHM or other techniques (*e.g.* fluorescence microscopy). As shown here, the inter-well variation of these parameters do not permit an accurate estimation of the cellular changes occurring after compound treatment. In addition to basic cell count and confluency measurements, the DHM signal also enables two approaches to monitor morphological changes induced by specific compounds. First, the analysis of the raw OPD signal providing a robust unbiased indicator of global morphological changes occurring in the cells and allowing to rapidly screen a large number of compounds and categorize them. Then, further CPA analysis allows distinguishing more subtle phenotypes such as those inducing the formation of vesicles; as could be achieved with fluorescence but without requiring any specific staining.

Another important advantage of DHM is that as no exogenous markers nor washing steps need to be added, repeated measurements can easily be done as soon as the tested compound is added, thus allowing to measure rapid kinetics occurring in the second time-range (see supplementary Fig. **4C** and [12]). In addition, each well can be its own control by comparing measurements before and after adding interfering compounds. This leads to an increase in precision of measurements and reduction of well requirements for a given experiment.

The label-free nature and the characteristic low light irradiation intensity of DHM allow continuous cell monitoring for long periods of time (three days, as shown in supplementary Fig. **3**). We monitored the evolution of the average OPD changes and “round” or “vesicle” phenotypes for the 24 first hours after addition of the tested compounds. Compared to simple endpoint measurements, more information can be extracted from these time-lapse experiments. Indeed, the onset of the compound response and the evolution of the signal over time generate a larger number of analyzed data points leading to more accurate and informative experimental results.

Other label-free optical techniques like DIC and PC also provide contrast from transparent specimens and a high level of cellular details allowing human qualitative analysis; however the absence of direct morphological relationships with the signal prevents automated cell segmentation and precise quantification. Some information can still be extracted from DIC and PC images using complex and case-to-case phenotype OPD restoration. TIE yields quantitative images related to the OPD of the cells, but it is directly dependent on the accuracy of the 3 BF-stacked images thus limiting the speed of the acquisitions. Only DHM combines the quantitative aspect, the resolution and the fast acquisition speed needed for automated tracking of global and sub-cellular morphological changes for screening applications.

## CONCLUSION

In summary we demonstrated the suitability of DHM, a quantitative noninvasive label-free microscopy technique, for automated phenotypic drug screening. Our results showed that its intrinsic quantitative phase contrast clearly highlights fine cellular morphological changes induced after compound addition. Moreover, thanks to the utilization of the OPD parameter for fast image analysis and the automated setup, we expect to include our DHM approach in the global strategy for screening campaigns for a broad range of applications. The inherent advantages of our label-free technology will also be further explored using a dual-mode microscope in combination with fluorescence microscopy for efficient biomarkers multiplexing with the aim to generate more informative HCS data.

## Figures and Tables

**Fig. (1):**
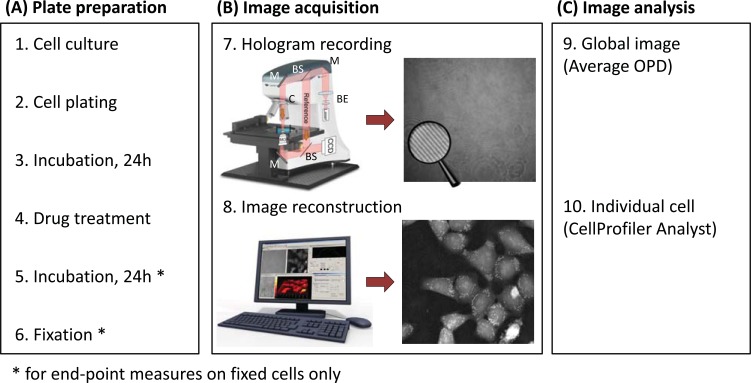
The Digital Holographic Microscopy (DHM) technology and experimental workflow. (A) Plate preparation procedure. (B) Image acquisition: First, a hologram (magnifying glass highlights the interference fringes) is recorded out of focus by a digital camera on a DHM T1001 system equipped with a motorized stage for automated multi-well plate experiments (7). Legend: M, mirror, BS, beam splitter, BE, beam expander, MO, microscope objective, C, condenser. Then, it is reconstructed by a computer to form an in-focus quantitative phase image (8). Contrast in DHM is provided by optical path length (OPL) variations in the specimen. For cell biology experiments, the measured optical path difference (OPD) is related to the thickness *d* and mean intracellular refractive index *n_c_* of the cultured cells, as well as to nm, the refractive index of the surrounding medium through Equation (1). (C) Image analysis is performed on the quantitative phase images using either on the global image (9) or on individual cell (10).

**Table 1.** DHM Performance: Z'-Factor Calculation for the Optical Label-Free Modalities Compared

		DHM				OPL from DIC				TIE from BF			
		HeLa		H9c2		HeLa		H9c2		HeLa		H9c2	
	**Z'-factor for: **	Doxo	Chloro	Doxo	Chloro	Doxo	Chloro	Doxo	Chloro	Doxo	Chloro	Doxo	Chloro
	**cell count **	**0.00 **	-0.33	-1.92	-3.76	-0.59	-3.29	-2.47	-3.11	-0.52	-6.82	-0.34	-10.6
	**confluency **	**0.37 **	-0.02	-0.75	-32.3	-0.48	-23.0	-4.86	-5.78	-0.19	-4.31	-2.06	-0.89
**DHM specific **	**average OPD **	**0.53 **	**0.37 **	**0.13 **	0.45	-1.81	-1.28	-0.41	**0.01 **	-0.11	**0.29 **	-0.88	-0.05
	**CPA analysis **	**0.88 **	**0.02 **	**0.46 **	0.76	**0.61 **	-2.04	-8.93	-10.3	**0.72 **	-0.74	**0.40 **	**0.75 **

Endpoint (24 h) calculated Z -factor for the round (doxorubicin, doxo ) and vesicle (chloroquine, chloro ) phenotypes in the HeLa and H9c2 cell lines for DHM, OPL restored from DIC and TIE (obtained from 3 BF images, described in the Imaging mode comparison section below). Z -factor above 0 are highlighted in green and below 0 in red. Cell count is per field of view (0.31 mm2). Confluency is the ratio of the cell surface per field area. Average OPD is the mean optical path length difference per surface area. CellProfiler Analyst (CPA) analysis is the percentage of cell expressing the round (for doxorubicin-treated cells) or vesicles (for chloroquine-treated cells) phenotypes. DHM specific denotes analysis that can only be conducted with a DHM instrument. n = 12-16 wells per conditions. The values used to calculate this table are provided in the supplementary data.

**Table 2.** Summary of the Advantages and Specificity of the DHM, DIC, PhC and TIE Techniques

	DHM	DIC	PhC	TIE
Label-free	+	+	+	+
Speed	+	+	+	-
Quantitative	+	-	-	-
Segmentablility	+	-	-	+
Ease of use	-	+	+	-
Cost	$$	$	$	$
