# Supplementary material for: Digital Holographic Microscopy: A Quantitative Label-Free Microscopy Technique for Phenotypic Screening
Source: Comb Chem High Throughput Screen. 2014 Jan;14(1):80–8. doi: 10.2174/13862073113166660062 (PMC3894694; doi:10.2174/13862073113166660062)
Supplement: Supplementary file 1 [file CCHTS-17-80_SD1.pdf]

SUPPLEMENTARY MATERIAL

Digital Holographic Microscopy: A Quantitative Label-Free Microscopy Technique for Phenotypic Screening

Benjamin Rappaz, Billy Breton, Etienne Shaffer, Gerardo Turcatti*

Biomolecular Screening Facility (BSF), Ecole Polytechnique Fédérale de Lausanne (EPFL), Lausanne, Switzerland

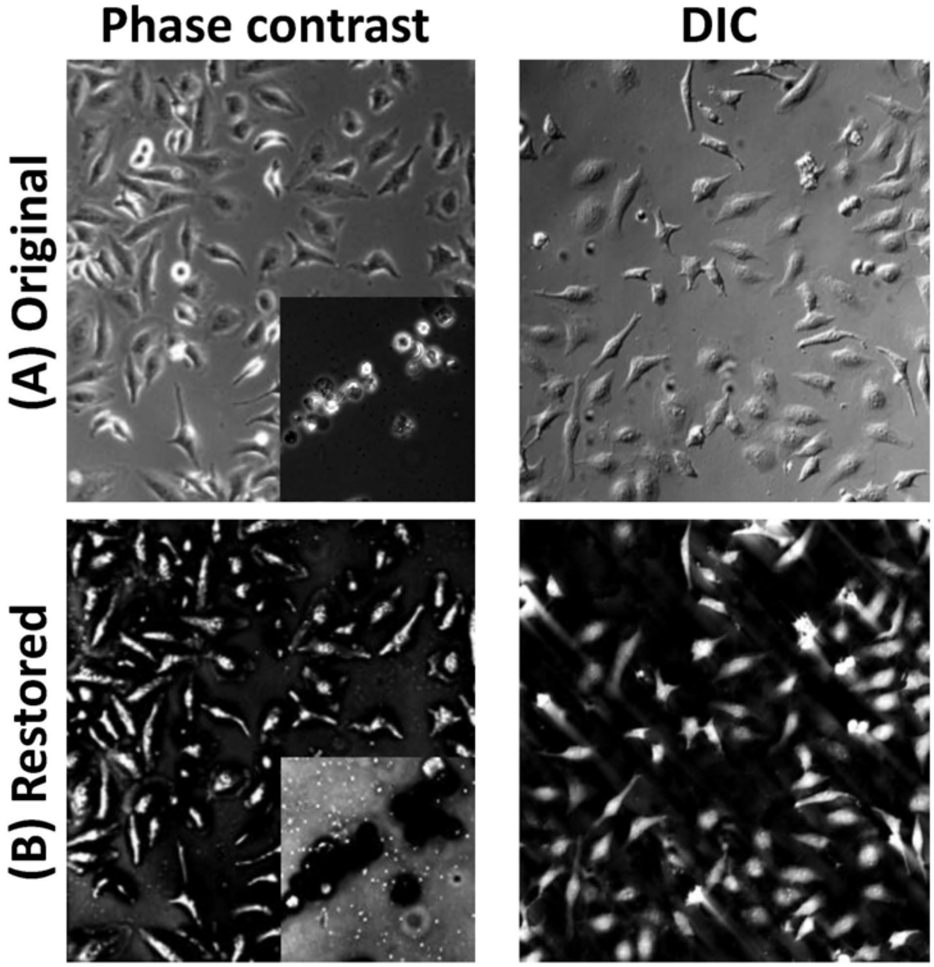

Supplementary Fig. (1). PC and DIC Image restoration. PC and DIC (row A) can be restored (row B) to generate pseudo-quantitative images where the signal is related to the OPD. DIC restoration produces only mild artifacts (cells have a small shadow parallel to the DIC shear angle) whereas PC restoration is only able to provide usable images with a precise phenotype, for instance if normal (flat) cells are correctly restored, rounded cells will "disappear" (as shown in the inset images, contrast adjusted).

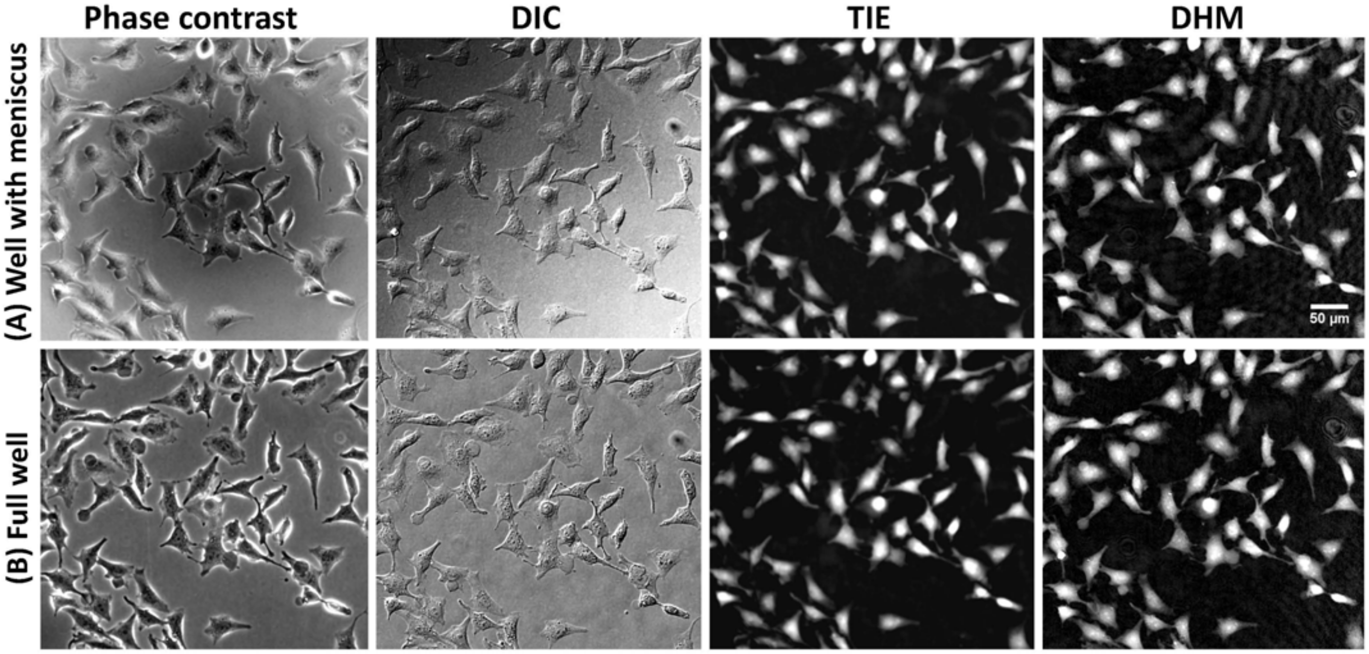

Supplementary Fig. (2). Image degradation due to the water meniscus. When the wells are normally filled (100 µl for a 96-well plate) a water meniscus forms (row **A**) and acts like a lens which degrades the image contrast. When the well is completely filled to the top of the side walls, a requirement not compatible with HCS, (row **B**) this effect disappears. This effect is absent with DHM, Bright Field and therefore TIE. Scale bar 50 µm.

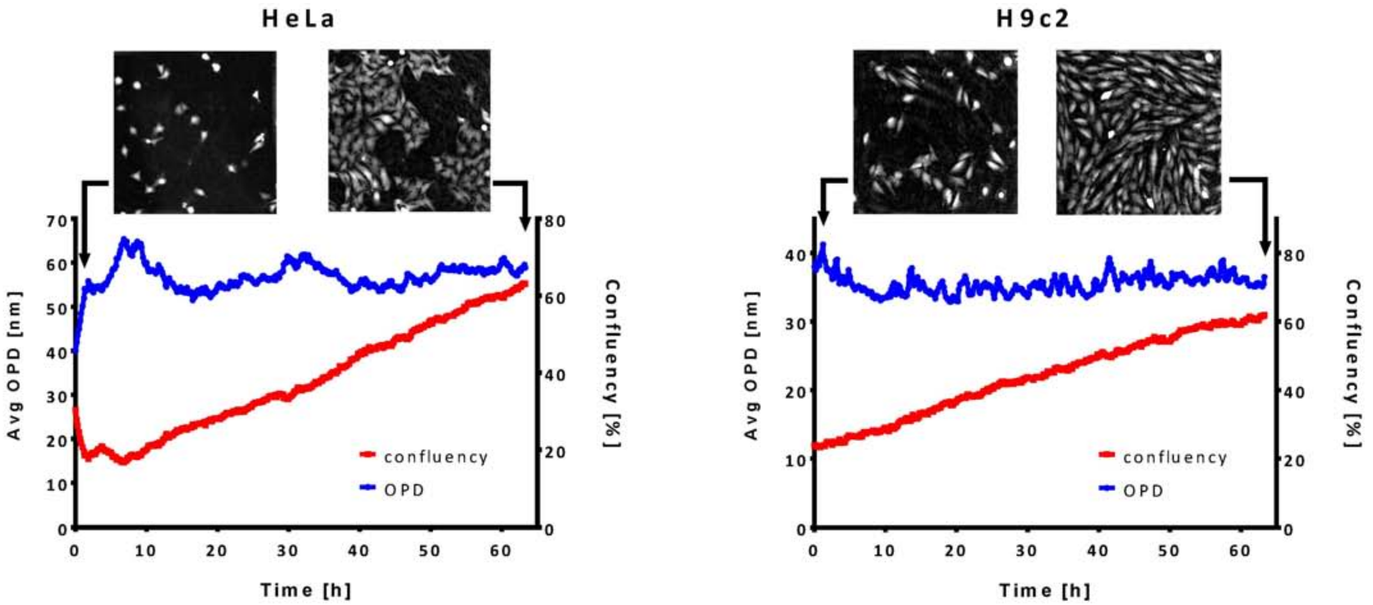

Supplementary Fig. (3). Effect of confluency on OPD measurement. Graphs shows the evolution of the measurement of confluency (red) and average OPD (blue) during a 65 hours time-lapse recording period with HeLa and H9c2 cells. We observe an early fluctuation of the OPD signal in the first few hours after plating (due to the sedimentation of the cells) and then a stable OPD for more than 2 days (much longer than the experimental time window). The lower measured confluency of H9c2 at the end of the experiment is due to the same threshold being used for both cells, thus giving a lower confluency for flatter cells (like H9c2).

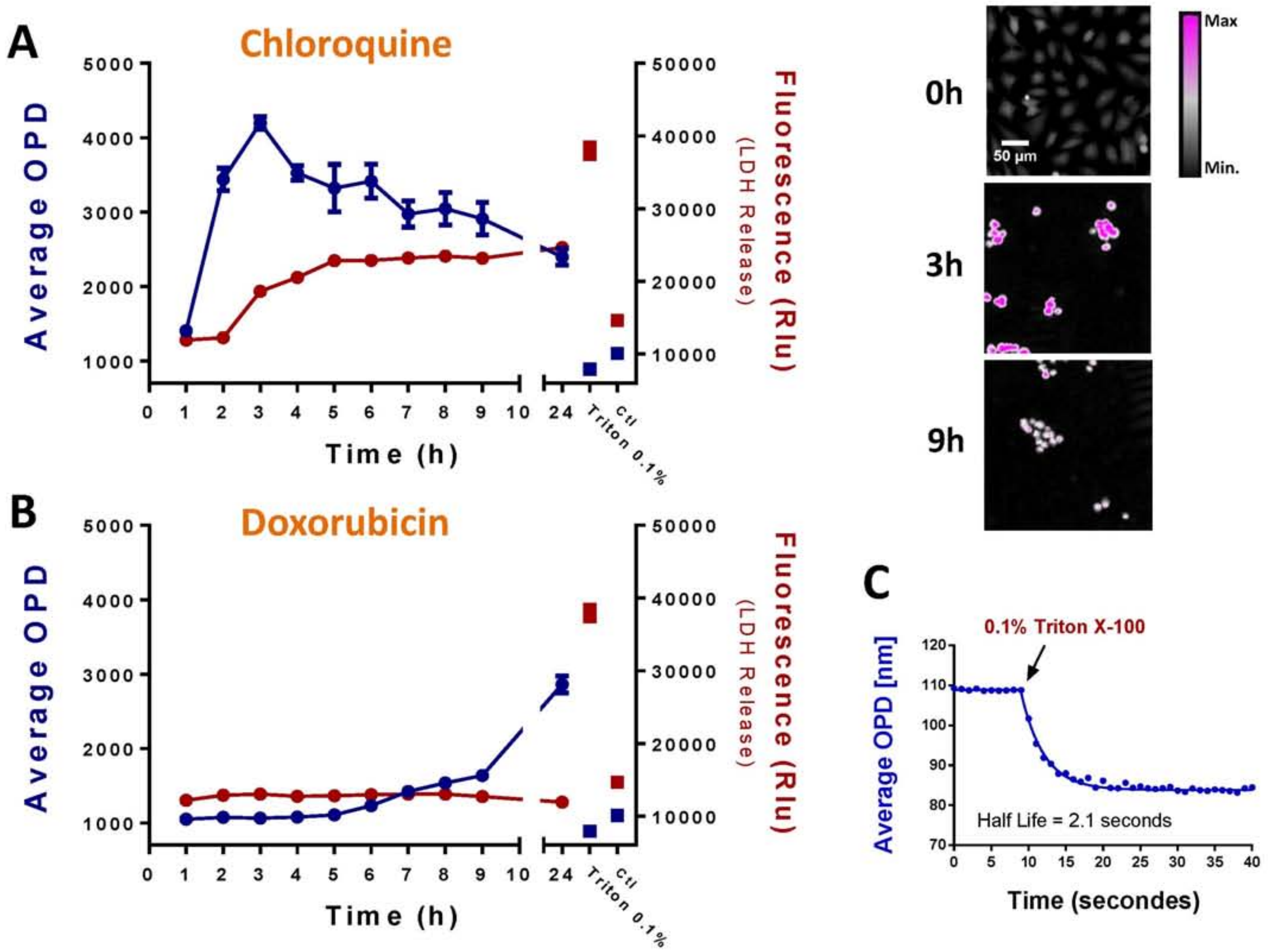

Supplementary Fig. (4). Membrane integrity assay. HeLa cells were plated in 96-well plates and treated at different time over 24 h with Chloroquine (1 mM) (**A**) or Doxorubicin (10 µM) (**B**). After indicated incubation time, DHM images were acquired and cells population average OPD signal were determined by a MATLAB R2012a image analysis algorithm at the same time total fluorescent signal were measured from Lactate Dehydrogenase Activity Assay kit (CytoTox-ONETM Homogeneous Membrane Integrity Assay, Promega). Increase in fluorescent signal corresponding to the release from cell to culture media of Lactate Dehydrogenase (LDH) is an indication of a lost of plasma membrane integrity. In the case of Chloroquine, the LDH increase activity start after 3 hours corresponding to the maximum signal measured in DHM. Whereas with Doxorubicin no apparent LDH increase in activity was observed. (**C**) HeLa cells were treated with 0.1% TritonX-100 and DHM signal was read online each second. TritonX-100 is a nonionic surfactant wildly use for cell lysis. Immediately after TritonX-100 addition, the average OPD decrease rapidly (with half live of 2.1 seconds).

Supplementary Table 1. DHM Performance: Z'-Factor Calculation for the Optical Label-Free Modalities Compared. Same Legend as Table 1 Except that the Values (Mean and Standard Deviation, SD) Used to Calculate the Z'-Factors are Provided.

			DHM															
			HeLa								H9c2							
	Analysis		ctrl		doxo		ctrl		chloro		ctrl		doxo		ctrl		chloro	
	(n = 12-16 wells)		mean	SD	mean	SD	mean	SD	mean	SD	mean	SD	mean	SD	mean	SD	mean	SD
	cell count	value	80.9	7.8	27.4	10.0			50.7	5.6	30.6	3.1	22.4	4.9			26.0	4.2
		Z'			0.00				-0.33				-1.92				-3.76	
	confluency [%]	value	17.8	2.0	3.0	1.1	same as doxo		8.8	1.1	5.6	0.7	3.0	0.8	same as doxo		5.4	1.1
		Z'			0.37				-0.02				-0.75				-32.3	
DHM specific	avg. OPD [nm]	value	115	2.3	213	13			167	8.7	83	3.2	159	19			149	8.7
		Z'			0.53				0.37				0.13				0.45	
	CPA analysis [%]	value	2.5	0.9	96.7	2.9	7.2	7.1	73.5	14.4	4.1	1.8	67.4	9.6	17.3	3.7	95.7	2.5
		Z'			0.88				0.02				0.46				0.76	

			OPL from DIC															
			HeLa								H9c2							
	Analysis		ctrl		doxo		ctrl		chloro		ctrl		doxo		ctrl		chloro	
	(n = 12-16 wells)		mean	SD	mean	SD	mean	SD	mean	SD	mean	SD	mean	SD	mean	SD	mean	SD
	cell count	value	149.3	9.3	103.2	15.1			135.3	10.7	93.2	5.9	75.5	14.6			84.0	6.7
		Z'			-0.59				-3.29				-2.47				-3.11	
	confluency [%]	value	20.4	3.0	8.8	2.7	same as doxo		19.6	3.1	6.1	2.3	4.2	1.4	same as doxo		7.7	1.3
		Z'			-0.48				-23.0				-4.86				-5.78	
DHM specific	avg. OPD [nm]	value	391	34	449	20			462	20	295	18	430	45			430	27
		Z'			-1.81				-1.28				-0.41				0.01	
	CPA analysis [%]	value	8.3	4.3	92.4	6.6	32.6	13.6	61.9	16.1	39.4	19.1	46.9	5.6	35.9	15.5	25.4	24.2
		Z'			0.61				-2.04				-8.93				-10.3	

			TIE from BF															
			HeLa								H9c2							
	Analysis		ctrl		doxo		ctrl		chloro		ctrl		doxo		ctrl		chloro	
	(n = 12-16 wells)		mean	SD	mean	SD	mean	SD	mean	SD	mean	SD	mean	SD	mean	SD	mean	SD
	cell count	value	230.2	27.8	111.0	32.4			205.0	38.0	113.4	8.3	68.5	11.8			119.1	13.7
		Z'			-0.52				-6.82				-0.34				-10.6	
	confluency [%]	value	11.0	1.7	3.8	1.2	same as doxo		9.2	1.6	3.4	0.6	2.3	0.6	same as doxo		5.4	0.7
		Z'			-0.19				-4.31				-2.06				-0.89	
DHM specific	avg. OPD [nm]	value	201	3.8	269	21			262	11	162	15	224	24			238	12
		Z'			-0.11				0.29				-0.88				-0.05	
	CPA analysis [%]	value	12.2	1.4	80.6	5.0	26.4	6.1	52.5	9.1	11.9	2.6	72.4	9.6	28.0	2.8	84.9	2.0
		Z'			0.72				-0.74				0.40				0.75	
